# More than gaming: Examining executive functions in esports players

**DOI:** 10.1371/journal.pone.0360942

**Published:** 2026-10-22

**Authors:** Serhat Yalçıner, Nahit Özdayı

**Affiliations:** Balıkesir University, Faculty of Sports Sciences, Department of Coaching Education, Balıkesir, Türkiye; İzmir Democracy University: Izmir Demokrasi Universitesi, TÜRKIYE

## Abstract

The main purpose of this study was to determine the differences in executive function performance levels of esports players who play First-Person Shooter (FPS) and Multiplayer Online Battle Arena (MOBA) game types compared to non-video game players (NVGPs). Other purposes of the study are to examine the relationships of executive function components and their relationships with in-game rank and total game play time variables. The study groups consisted of ninety volunteer participants, all of whom were male young adults between the ages of 18 and 34, 30 of whom actively played FPS video games, 30 of whom actively played MOBA video games, and 30 of whom NVGPs. Corsi Block-Tapping Test (CBT), Wisconsin (Berg) Card Sorting Test (WCST), and Stroop Test Çapa Form (ST) were used as data collection tools. According to the findings of the study; FPS players had significantly higher scores than NVGPs (p < 0.001, F = 8.55) in CBT and less error percentage than the NVGPs (p < 0.01, F = 5.58) and MOBA players had significantly less interference time than NVGPs in the ST (p < 0.001, F = 7.66). In addition, there was a significant negative correlation between CBT and ST interference times for all players (p < 0.05; r = −0.28) and a significant negative correlation between in-game skill and ST interference times for FPS players (p < 0.05; r = −0.41). These findings suggest that digital video game experience shapes cognitive profiles based on genre-specific demands; while FPS gameplay primarily enhances visuospatial working memory capacity, MOBA gameplay fosters stronger inhibition control, with higher in-game skill level reflecting superior inhibitory control in FPS gameplay, and relationship of visuospatial working memory and inhibition components demonstrating an underlying interrelated structure.

## 1. Introduction

Esports are events organized by different leagues and tournaments, around competitive video games, and are usually supported by various business organizations, between professional or amateur teams [1]. Esports, which stands out as a rapidly growing industry carried out through competitive video games, emerged in the late 1990s [2]. Today, esports, which stands out as an intersection of video games and professional sports, has become a major industry with corporate federation structures in 152 countries around the world [3] and millions of followers and fans. The popularity of these competitive games played in a virtual environment (playing, watching, etc.) is increasing rapidly [4] and it is striking that they have many similarities with traditional sports [5]. Given that video gaming has become widespread and is a topic of interest to many people, it is important to scientifically determine its positive or negative cognitive effects in order to protect oneself from these negative effects and consciously benefit from the positive ones.

Although there are many different types of video games, they are not all included in the same classification because they do not allow real-time competition [6,7]. First-person shooters (FPS) and Multiplayer Online Battle Arena games (MOBA), which are among the most popular video game genres in the world [8], differ from each other in many ways in terms of the different skills they require, the different types and levels of stimuli they have, their perspectives and purposes [9]. At this point, it is thought that it is important to examine competitive video games by taking into account the different features of the games. Are esports players, who are exposed to a large number of stimuli in a short time during the game (the opponent’s attack, defense and tactical moves, match positions, etc.) and who have to organize their behaviors by processing these stimuli in order to dominate their opponents and be successful in the games, different from non-video game players in terms of executive function skills? Based on this question mark, it has been wondered whether executive function skills, which are of great importance in almost all areas of human life [10–12] are related to playing video games.

In esports, cognitive processes that help maintain long-term performance and play an important role in achieving satisfactory results are especially executive functions [13,14]. Many of these games require players to focus on multiple stringent tasks, pushing the limits of attentional control, working memory, and executive function capacities [15]. Managing many different factors simultaneously during esports games (such as map, environmental control, tactics, communication, aiming) creates a certain level of cognitive load on the player. To manage all these variable factors and achieve success by making the right decisions, the esports player needs high cognitive skills [16]. Executive functions are the skills that enable the organization of goal-oriented behaviors [17].

Players may need high cognitive skills to be successful in video games [18,19], and esports can contribute to the development of motor and cognitive skills [20,21]. In action video games (including FPS games), players must control and execute multiple simultaneous tasks with high processing speed [22]. In particular, increased experience in action video games has positive effects on executive functions [23]. Players must always update important information necessary for game progress [24] and adapt their actions to constantly changing task conditions [25]. It is seen that current study results in the literature report the positive effects of video games on cognitive skills [18,22,26–38].

The focus of the study was on the possible effects of playing FPS and MOBA games on these executive functions within the framework of the three-category executive function model consisting of working memory, cognitive flexibility, and inhibition components [39,40]. The fact that many recent studies in the literature provide evidence on the effects of playing various video games on general cognitive skills [21,26,33,35,41] and that individuals’ executive functions can be improved through gaming experience or training [42–45] motivated the investigation of these effects in more detail. This study provides an original contribution to the emerging literature on cognitive skills in esports, primarily regarding differences in executive function levels between esport video game players and non-video game players, the relationships between known-to-be-related executive function components [46,47] in esports players, and the relationships between esports players’ in-game skill level and total play time.

This study was designed as a cross-sectional study to examine the relationships between playing FPS and MOBA esports games, in-game rank, and total playtime with executive function skills, to provide foundational evidence for longitudinal studies on the effects of esports participation on executive function skills, and to conduct preliminary research for the development of more rational and scientific esports training models in the future.

## 2. Methods

### 2.1. Study participants

The research group of the study consisted of a total of ninety male young adults between the ages of 18–34 volunteer participants, 60 of whom were had no known cognitive disorders or color vision defects, were at least high school graduates, were right-handed, actively played First-Person Shooter/FPS (Valorant, Counter-Strike: Global Offensive/Counter-Strike 2) and Multiplayer Online Battle Arena/MOBA (League of Legends and DOTA2) video games and had at least 1000 hours of highly experienced in these games, were native Turkish speakers, and thirty participants stated that they had not played any video games regularly and more than 200 hours and met the other participation criteria [48]. Gay, Mills, and Airasian [49] suggest a minimum of 30 participants per group as a general sample size criterion for correlational, causal-comparative, and experimental research designs. Accordingly, it was deemed appropriate for this study to have 30 participants in each group, resulting in a total sample size of 90 individuals. When the characteristics of the study groups presented in Table 1 are examined, it is seen that the groups have similar characteristics in terms of independent variables (p > 0.05).

**Table 1:** Characteristics of the study groups ($\overset{―}{𝐗}$±SD).

Variables	Groups	n	$\overset{―}{𝐗}$±SD	F/t	p
**Age (years)**	**FPS**	30	23.13 ± 5.15	0.82	0.47
	**MOBA**	30	22.43 ± 4.35		
	**NVGP**	30	21.70 ± 3.33		
**Education (years)**	**FPS**	30	14.70 ± 2.58	0.37	0.69
	**MOBA**	30	14.20 ± 2.44		
	**NVGP**	30	14.33 ± 1.89		
**In-game skill level** **(percent)**	**FPS**	30	87.81 ± 16.52	1.58	0.12
	**MOBA**	30	81.06 ± 16.59		
**Total play time (hours)**	**FPS**	30	3850 ± 2840.08	−0.93	0.36
	**MOBA**	30	4620 ± 3526.39		

FPS: First-Person Shooter player, MOBA: Multiplayer Online Battle Arena player, NVGP: Non-video game player.

### 2.2. Data collection tools

#### 2.2.1. Participant information form.

It is the form that contains information about the participants’ age, esports game, in-game skill level (rank), total playing time and education status.

#### 2.2.2. Corsi block-tapping test.

It is a classic visuospatial working memory test. In this study, the Psychological Experiments Language (PEBL 2.1) program, which includes a computerized version of Corsi, was used [50]. Three trial applications are performed before the scored test. The scored test starts with a series of 2 (each series is performed 2 times. After each series of 2 correctly answered, the boxes in the series increase by one. The participant is allowed to give only one incorrect response for each series. If two incorrect responses are given in the same series, the test ends [51]. The total score obtained from this test shows the level of the individual’s visuospatial working memory capacity.

#### 2.2.3. Wisconsin (Berg) Cart Sorting Test-64.

In this study, the Psychological Experiment Language (PEBL 2.1) program, which includes a 64-card computerized version of the Wisconsin (Berg) Card Sorting Test (WCST), was used [52]. The standard WCST consists of a 128-card deck. As a result of the study conducted for the shortened 64-card version, it was stated that there were high correlation values between the two versions (r = 0.77–0.87) and that the 64-card version was a usable measurement tool [53]. Each card contains a different combination of four shapes, four colors, and one of four quantities. Four key cards are displayed at the top of the screen as a guide to help determine which of the four decks the open card of the deck is sorted into. One card is revealed at a time, and this card is matched with the cards according to a random rule for a particular set. The sorting rule changes after each set (also unknown to the person being examined). The new rule must be discovered through trial and error based on the feedback received after each card is sorted. After a card is sorted, the participant is given feedback on whether the card is sorted correctly (according to the current rule). This process continues until the participant has sorted all 64 cards [53]. The total percentage of errors obtained from this test indicates the level of the individual’s cognitive flexibility capacity.

#### 2.2.4. Stroop Test Çapa version.

The Stroop Effect [54] is a typical inhibition task [55]. In the study, the Çapa Form of the Stroop Test [56] was used to measure the inhibition variable. This form is the adaptation of the Stroop Form developed by [57] by the Istanbul Faculty of Medicine (Çapa) Neuropsychology Laboratory. The Stroop Test Çapa Form consists of two stimulus cards, each consisting of a total of 60 items in a 6x10 arrangement and a total of 3 different colors and color names (red, blue, green). The first card contains small rectangles in red, green, and blue. On the second card, the color names are written in incompatible ink colors (for example, the word red is written in green and the word green is written in blue). The test has an application consisting of three stages. In the first part, the participant is asked to say the colors as quickly as possible, from left to right. If there is any visual impairment at this stage, the test is terminated. In the second part, the participant is asked to read the color names written in incompatible ink colors as quickly as possible. In the third part, the participant is expected to say the ink color (red, blue or green) instead of reading the color names. All three parts are timed and the time is recorded when the task is completed. The interference resistance (resistance to disruptive effects) time is obtained by subtracting the second part time from the third part time [56]. The shorter this time, the better the level of inhibition.

### 2.3. Data collection procedure

The necessary ethical approval for this study was obtained from Balıkesir University Social and Human Sciences Ethics Committee (2023/04). Participants were recruited between [01.12.2023] and [01.06.2024]. All participants provided written and signed informed consent prior to participation in the study. All tests were demonstrated and explained to participants in detail using a standard procedure. The tests did not begin until it was ensured that participants understood them. Participants did not attempt to take the tests. The tests were applied to all participants in the same order and with the same computer/mouse.

### 2.4. Statistical analysis

SPSS 30.0 (Statistical Package for Social Sciences) program was used in the analysis of the data set. Before statistical analysis, whether the data had a normal distribution was evaluated through skewness and kurtosis (±1.5) values [58]. The analyses of the data determined to have a normal distribution were carried out through parametric hypothesis tests. One-Way ANOVA was used in the comparison of test values between groups, and Tukey HSD [59] test was preferred as a post-hoc test because the number of people in the groups was equal and the assumption of homogeneity of variances was met. Additionally, the significance level of post hoc test results was evaluated at 0.017 (0.05/3), with Bonferroni correction applied to prevent type 1 error. While Pearson Correlation Analysis was used in the relationship analysis, Simple Linear Regression Analysis was used in the analysis of the predictive role of the variables found to have a significant relationship.

## 3. Results

### 3.1. Differences in executive function levels of study groups

The results of the Analysis of Variance (One-Way ANOVA) conducted to evaluate the differences between the FPS, MOBA, and Non-Video Game Player groups in the values obtained from the tests conducted to measure executive function levels are presented in Table 2. According to Table 2, it was determined that there was a statistically significant difference between the groups in each of the executive function test values (p < 0.05). According to the Tukey-HSD post-hoc analysis results used to determine which groups the difference was between, it was determined that the FPS player group had statistically significantly higher values than the NVGP group in CBT total score, the NVGP group had statistically significantly higher values than the FPS player group in WCST total error percentage, and the NVGP group had statistically significantly higher values than the MOBA player group in ST interference duration (p < 0.05). The effect sizes of the group independent variable on the dependent variables were found to be a “large” effect (η2 = 0.16) for the CBT total score variable, a “medium” effect (η2 = 0.11) for the WCST total error percentage variable, and a “large” effect (η2 = 0.15) for the ST interference duration variable.

**Table 2:** One-Way ANOVA results regarding the differences between groups in executive function test values.

Dependent Variable		Source of Variance	df	Sum of Squares	Mean Square	F	p	Tukey-HSD	η2
**Corsi Block-Tapping Test**	**Total Score**	**Between Groups**	2	7530.96	3765.48	8.55	<0.00	a > c***	0.16
		**Within Groups**	87	38310.17	440.35				
		**Total**	89	45841.12					
**Wisconsin Card Sorting Test-64**	**Total Error (percent)**	**Between Groups**	2	128.07	64.03	5.58	<0.01	c > a**	0.11
		**Within Groups**	87	998.42	11.48				
		**Total**	89	1126.48					
**Stroop Test Çapa Version**	**Interference Time** **(seconds)**	**Between Groups**	2	1350.94	675.47	7.66	<0.00	c > b**	0.15
		**Within Groups**	87	7674.89	88.22				
		**Total**	89	9025.83					

**: p < 0.01, ***: p < 0.001. df: degrees of freedom, a = FPS, b = MOBA, c = NVGP.

### 3.2. Correlations of executive function components in esports players

We performed a Pearson correlation analysis to examine relationships among executive function test scores in all esports players (n = 60). A statistically significant negative relationship was found between CBT total score and ST interference time (p < 0.05; r = −0.28), and the predictive role analysis was performed using simple linear regression analysis in Table 3. It was found that there was no statistically significant relationship between other executive functions (p > 0.05, r = −.170; −.080). When the regression analysis presented in Table 3 was examined, it was determined that the CBT total score as a predictor variable in all players was a significant predictor of the ST interference duration (F(1, 58)=4.82; p < 0.05) and explained 8% of the variance (R^2^ = 0.08), and a one-unit increase in the CBT total score caused a 0.12-unit decrease in the ST interference duration (B = −0.12).

**Table 3:** Simple linear regression analysis results regarding the predictive value of CBT total score for ST interference time.

Variables	B	SE	β	t	p
Constant (ST)	37.49	3.82	–	9.83	0.00*
CBT Total Score	−0.12	0.05	−0.28	−2.20	0.03*

*Durbin-Watson: 1.92, F*_*(1, 58)*_ *=4.82, R=0.28, R* ^*2*^ =0.08, *: p<0.05. SE: Standard Error, ST: Stroop Test Çapa Version, CBT: Corsi Block-Tapping Test.

### 3.3. Relationship of executive functions with in-game rank and total play time

Pearson correlation test was performed on the relationship between in-game ranking and total game time with executive function tests for FPS (n = 30), MOBA (n = 30), and all players (n = 60). A statistically significant negative correlation was found between in-game skill levels of FPS players and ST interference times (p < 0.05; r = −0.41). No statistically significant correlation was found between other executive functions and in-game rank and total game time (p > 0.05). According to Table 4; it was determined that the in-game skill level as a predictor variable was a significant predictor of the ST interference time (F(1, 28)=5.50; p < 0.05) and explained 16% of the variance (R^2^ = 0.16), and a one-unit increase in the in-game skill level caused a 0.28-unit decrease in the ST interference time (B = −0.28).

**Table 4:** Simple linear regression analysis results regarding the predictive value of in-game rank on ST interference time in FPS players.

Variables	B	SE	β	t	p
Constant (ST)	55.99	10.56	–	5.30	0.00*
IGR	−0.28	0.118	−0.41	−2.35	0.03*

*Durbin-Watson: 2.29, F*_*(1, 28)*_*=5.50, R = 0.41, R*^*2*^ = 0.16, *: p < 0.05. SE: Standard Error, ST: Stroop Test Çapa Version, IGR: In-game rank

## 4. Discussion

This study mainly aimed to determine the executive function skill levels of FPS and MOBA players (visuospatial working memory, cognitive flexibility and inhibition) and to examine the differences between them and non-video game players. In addition, the other aims of the study were to examine the relationships between the executive function components and the in-game skill level, total game time variables and their predictive roles. The main findings were as follows: 1) FPS players have higher visuospatial working memory performance than NVGPs; 2) NVGPs have lower cognitive flexibility performance than FPS players; 3) MOBA players have higher inhibition capacities than NVGPs; 4) Visuospatial working memory performances of esports players are positively correlated with their inhibition performances; 5) FPS players’ in-game ranks are positively correlated with their inhibition performances.

### 4.1. Differences in executive functions by game genre and game playing status

#### 4.1.1. Visuospatial working memory.

It is thought that the reason why FPS players have better visuospatial working memory capacities than NVGPs is that FPS games have some advanced cognitive visuospatial requirements due to their dynamics [26,41,60] and that the visuospatial working memory levels of those who play these games develop over time to adapt to these requirements. In MOBA games, players have a better command of the map and the environment compared to FPS games, and since they play from a third-person perspective and have wider viewing angles, they can realize the situations that may create danger in the game earlier. In this case, it is thought that FPS players who play from a first-person perspective may have more developed visuospatial working memory capacities compared to MOBA players because they have to struggle with a higher level of arousal and use their visual attention more throughout the match in order to respond quickly to instant developments they encounter. On the other hand, due to the cognitive competence and activation levels that FPS games demand from players, FPS players, compared to MOBA players, need to visually record more detailed maps and areas where opponents may be, remember these records and use them in subsequent games, quickly scan the environment during the game to become aware of constantly changing situations (opponents, obstacles, etc.), and exhibit fast and accurate behaviors are important for success. It can be said that all these reasons require FPS players to use their visuospatial memory capacity more, and therefore, the development is greater than MOBA players and NVGPs.

In line with the study results; Mayer [61] reported that students who played action video games (including FPS games) showed greater increases in perceptual attention than students who played other types of games and did not play games. Barlett et al. [62] reported that video games contribute to the improvement of visual processes such as visualization and spatial skills and increase cognitive performance through more efficient information processing; Blacker and Curby [63] reported that action video game players had better short-term visuospatial working memory levels than NVGPs; Chisholm et al. [64] and Hubert-Wallander et al. [65] reported that action video game players had better visual attention levels than those who played other games; Colzato et al. [66] reported that the FPS game group exhibited faster and more accurate working memory performance than the group that did not play any games. When meta-analysis studies in the literature are examined, it has been stated that video games significantly improve information processing [36], especially action video game training has positive effects on cognitive skills [67], and spatial cognition, which is a part of visuospatial working memory, can be improved through action games [26]. However, Green and Bavelier [29] reported that those who play action video games are better at scanning and detecting information in visual environments than those who do not play games. Toril et al. [68] conducted a control group study to evaluate the effects of video games on visuospatial working memory in healthy elderly people and had the experimental group play video games for 15 sessions (1 hour each). As a result of the study, they observed significant improvements in the experimental group in the Corsi Block-Touch and jigsaw puzzle tasks they performed to evaluate visuospatial working memory. In line with these findings, it can be said that education through video games can serve as an effective intervention to improve working memory and other cognitive functions not only in the young population but also in the elderly.

#### 4.1.2. Cognitive flexibility.

It can be said that the reason why FPS players have better cognitive flexibility levels than NVGPs is due to the cognitive adaptation process that occurs as a result of intensely entering into these situations that require cognitive flexibility and multitasking due to the necessity of managing multiple tasks simultaneously, creating/changing strategies quickly and adapting to changing conditions during competition, which is present in FPS game types. On the other hand, it is thought that NVGPs may not have reached a cognitive flexibility capacity as good as gamers because they are not exposed to stimuli that requires this level of intensity of cognitive flexibility in their daily lives as much as gamers.

In line with the study results, Colzato et al. [66] stated that action games, especially FPS games, contribute to the development of cognitive flexibility. Green and Bavelier [69] reported that playing action video games provides significant improvements in basic perceptual skills and cognitive flexibility, and Hoseini et al. [32] reported that playing 3D games has a significant positive effect on cognitive flexibility. In systematic review and meta-analysis studies, cognitive flexibility has been associated with game performance [26,35,70,71]. As a result of the study conducted by Imanian et al. [33], in which they examined the cognitive differences that occurred as a result of the process by having NVGPs play single-player and multiplayer games for eight weeks, they observed that there was no significant change in the cognitive flexibility levels of those who played single-player games, while there was a significant increase in the cognitive flexibility levels of individuals who played multiplayer games. On the other hand; Podlogar and Podlesek [72] reported that although action video game players switched between tasks faster than NVGPs, they did not differ in terms of cognitive flexibility.

#### 4.1.3. Inhibition.

It is thought that the reason why MOBA players have better interference times than NVGPs in the inhibition task is due to the long-term strategic plans that extend until the end of the game in MOBA games, acting for the benefit of the team instead of instant reactions and managing many tasks in coordination with the team being at the forefront in order to win the competition, and the potential for contributing to the development of a high-level inhibitory control mechanism in players. Although there is no significant difference between FPS players and MOBA players, it is thought that the reason why MOBA players have better interference times than FPS players is due to the fact that in FPS games, reacting with mostly fast reflexes is spread throughout the game, and this habit has the potential to negatively affect the inhibition level. In terms of game dynamics, in competitive modes where teams of five players face off in FPS games, the probability of a single player winning the game for the team is higher than in MOBA games. Because in FPS games, when it is assumed that each player is on equal terms, winning the round can also be possible with a talented player’s high-level single-player performance. This situation is very rare at the professional level, but more common at the amateur level, and players may be more prone to individual skill and performance to show themselves when the conditions are right. Despite this, the fact that FPS players have better interference time than NVGPs also shows that the requirements of FPS games improve the inhibition capacity, although not as much as in MOBA games. Because although team play is important in both game types, team cooperation of players is more prominent in MOBA games than in FPS games, and as in the example given for the FPS game, it is almost impossible for the team to win the match by defeating all the minions, towers and players alone. It is thought that this situation may be due to the fact that MOBA players tend to act together with the team instead of acting individually, keeping their sudden reactions under more control in order to win the match, and that adapting to team play is a necessity in order to win. These situations described are observed very differently at the amateur level and the professional level. Professional esports teams prioritize team coordination for both FPS and MOBA games and create game tactics based on this. However, since amateur players generally do not enter into a systematic training process as a team, individual action is more frequently observed in these situations. Considering all these details, it is thought that the inhibition skills of FPS and MOBA players need to be investigated more comprehensively.

When the relevant literature is examined; Hoseini et al. [32], in their study examining the effects of playing 2D and 3D video games on cognitive parameters, found that playing these games positively and significantly affected inhibition skills; Deleuze et al. [73] stated that FPS players exhibited faster reaction times than MOBA players, but had lower control over inhibition. However, it has been reported that video game players can improve their ability to filter out unnecessary or irrelevant stimuli (inhibition) through the development of attention function [25,64] This situation has also been supported by neuroimaging studies showing that differences in brain activation affect cortical functioning after long-term video game play. In this context, Bavelier et al. [74] reported using functional magnetic resonance imaging (fMRI) that, in a condition involving increasing distractions, action game players showed a slight increase in activation levels in the fronto-parietal regions associated with attention processing [75] as distractions increased. These results suggest that action video game players are neurally more efficient in using attention, with less activity increase observed in cortical activity, and thus the cortical regions that provide attention can filter out distracting information more easily. Mishra et al. [76] reported that video game players had better inhibition levels than NVGPs in a study examining the inhibition skills of video game players, and this was supported neurophysiologically by event-related potentials (ERPs) associated with perceptual discrimination and decision making. On the other hand, there are also studies indicating that the results regarding inhibitory control in studies on video gamers are not conclusive [77,78].

#### 4.1.4. Relationship between executive functions in esports players.

It is thought that the positive correlation between visuospatial working memory capacities and inhibition capacities in all esports players is due to the fact that players have to process rapidly changing visuospatial information quickly and effectively in order to be successful in games. The increase in visuospatial working memory activation due to the need to remember important information about the opponent with in-game environmental changes, the need for inhibition skills to focus their attention/prevent distraction, and the intensive cooperation of these two executive functions to manage in-game dynamics such as fast decision making and creating/maintaining the right strategy can also be shown as factors that may affect this situation.

Due to the limitation of the esports literature regarding this result, based on the general executive function literature, it is known that executive function components work in an integrated manner and are related to each other [46,47]. Wiebe [79] provided evidence that the development of different executive function processes may be reciprocally related, for example, improvements in working memory may create changes in inhibition. While examining the structure of executive functions in adults, Miyake et al. [40] found three separate but related structures (working memory, cognitive flexibility, and inhibition), and in examining the relationship between these three structures, they stated that inhibition and working memory were the structures with the highest positive relationship dimension. Beattie et al. [80] found that children with better attention and inhibition skills have better visuospatial working memory capacities; Szucs et al. [81] reported that potential problems in visuospatial processing and attention functions during the developmental process in children are likely due to working memory and inhibition disorders.

#### 4.1.5. Relationship of executive functions with in-game rank and total play time.

It has been observed that FPS players’ in-game skill levels and their inhibition capacities are positively correlated. Since FPS games have a very variable and fast-paced structure and expect players to make quick decisions, the probability of making mistakes also increases. Since FPS players with high in-game skill levels, and therefore more in-game dominance and experience, have engaged in this quick decision-making process more, it is thought that it is an expected situation that the increase in their stimuli processing and behavioral inhibition capacities will move together with the in-game skill level. In short, as FPS players’ in-game skill levels increase, their inhibitory control increases even more, and as their in-game skill levels increase, they can better block distractions in working memory in decisions made quickly. In line with the study results; Zhang et al. [82] examined the performance of action video gamers and strategy video gamers in inhibiting distracting stimuli in tasks with different levels of perceptual load, and stated that there was a significant difference in task performance between the two types of players under high perceptual load, and that action video game players were better at inhibiting distracting stimuli under high perceptual load. Similarly, Hutchinson et al. [83] examined the effects of 10 hours of various game playing experiences and concluded that individuals exhibited significantly better inhibition skills and reaction time after FPS game experience. When some studies containing results in terms of neural activity were examined, Hazarika et al. [84] reported that increased experience in action video games improves inhibitory control mechanisms and that neural activity in the alpha frequency band may be an indicator of active inhibitory control.

### 4.2. Limitations

Several methodological limitations of the present study should be acknowledged. First, regarding gender and age, our sample was exclusively composed of male participants aged 18–34. While this approach was deliberately chosen to control for gender- and age-related confounding variables in visuospatial processing and executive control, it restricts the external validity of our findings. Consequently, the results cannot be directly generalized to female esports players or broader populations, who may exhibit different cognitive responses to video game play. Second, in terms of game-specific limitations, the study evaluated only FPS and MOBA genres. Although these genres heavily tax real-time decision-making, working memory, and visuospatial attention, they represent distinct cognitive profiles compared to other popular genres such as Real-Time Strategy (RTS), Fighting, or Turn-Based Strategy games. Therefore, the observed cognitive patterns may be specific to the fast-paced, high-load mechanics of FPS and MOBA titles rather than gaming in general. Finally, the cross-sectional design of this study inherently restricts our ability to infer causal relationships; thus, it remains unclear whether gameplay actively enhances executive functions or whether individuals with higher baseline cognitive abilities naturally gravitate toward these gaming genres.

#### 4.2.1. Methodological considerations.

First, the decision to include exclusively male participants was a deliberate methodological strategy designed to enhance internal validity. By restricting the sample to males, we effectively controlled for potential gender-related neurobiological and cognitive confounding variables, ensuring that observed differences in executive function could be attributed primarily to gaming status rather than biological sex. While it minimizes internal noise, it restricts the external validity and generalizability of our findings. Given the growing representation of female players in recreational gaming ecosystems, future research should incorporate balanced female cohorts to examine whether video-game-induced cognitive adaptations manifest similarly across genders. Second, the structural profile of amateur players provides a unique scientific vantage point compared to elite professional esports athletes. Elite players are subject to rigorous, highly regimented training schedules often exceeding eight hours daily and undergo intense selection processes that may select for naturally superior baseline cognitive abilities. Consequently, elite cohorts frequently display a “ceiling effect,” where further cognitive modulation is difficult to isolate. In contrast, amateur players engage in recreational gaming with lower, more varied training volumes. Examining amateur players against non-gamers allows us to evaluate whether non-professional, everyday gaming activity is sufficient to induce measurable cognitive shifts in executive functions such as working memory, task-switching, and inhibitory control. Our findings demonstrate that even non-elite, recreational engagement can confer cognitive advantages over non-gaming controls, highlighting the potential accessibility of video games as a medium for general cognitive stimulation.

### 4.3. Suggestions

A study with a design like this study, which includes groups of amateur esports players with various in-game skill levels, can be conducted with a sample that includes professional esports players. In this way, the differences required to be a professional esports player can also be revealed. In addition, the effects of other game types on executive function skills can be examined and the literature on the cognitive effects of game types can be contributed to. Finally, this study focused solely on behavioral performance measures derived from cognitive tests and did not directly record neurophysiological or neuroimaging data (such as EEG or fMRI). Consequently, while our findings demonstrate clear changes in behavioral performance, inferences regarding specific neurobiological and physiological pathways remain indirect. Future research integrating behavioral tasks with real-time neuroimaging is recommended to directly map the physiological processes underlying these cognitive differences.

## 5. Conclusion

This study comprehensively revealed the distinctive effects of different game types and gaming experiences on cognitive processes. The findings showed that FPS players had higher visuospatial working memory capacities than both MOBA players and non-videogamers. Additionally, the fact that the non-gaming group’s cognitive flexibility levels were significantly lower than both player groups suggests that interactive and fast-paced digital games may support cognitive flexibility. The finding that MOBA players’ inhibition capacity was higher than that of non-gaming individuals indicates that such games can strengthen attention control and response inhibition processes. When all esports players participating in the study were evaluated, a positive relationship was found between visuospatial working memory capacity and inhibition capacity, and working memory was determined to be a significant predictor of inhibition. This result indicates that executive functions mutually support each other in the context of esports. Furthermore, the in-game skill level of FPS players was found to be positively related to inhibition capacity and emerged as a significant predictor of inhibition. This situation shows that game performance is fueled not only by motor skills but also by high-level cognitive control processes. Overall, the findings reveal that different types of digital games affect distinct cognitive mechanisms in unique ways and that executive functions play a critical role in esports performance.
